# Advancing scoping study methodology: a web-based survey and consultation of perceptions on terminology, definition and methodological steps

**DOI:** 10.1186/s12913-016-1579-z

**Published:** 2016-07-26

**Authors:** Kelly K. O’Brien, Heather Colquhoun, Danielle Levac, Larry Baxter, Andrea C. Tricco, Sharon Straus, Lisa Wickerson, Ayesha Nayar, David Moher, Lisa O’Malley

**Affiliations:** 1 Department of Physical Therapy, University of Toronto, 500 University Avenue, Room 160, Toronto, Ontario M5G 1V7 Canada; 2 Institute of Health Policy, Management and Evaluation (IHPME), University of Toronto, 155 College Street, Suite 425, Toronto, Ontario M5T 3M6 Canada; 3 Rehabilitation Sciences Institute (RSI), University of Toronto, 500 University Avenue, Room 160, Toronto, Ontario M5G 1V7 Canada; 4 Department of Occupational Science and Occupational Therapy, 500 University Avenue, Room 160, Toronto, Ontario M5G 1V7 Canada; 5 Bouve College of Health Sciences, Department of Movement and Rehabilitation Sciences, Northeastern University, 360 Huntington Avenue, Boston, MA 02115 USA; 6 Community Member and Knowledge User, Halifax, Nova Scotia Canada; 7 Li Ka Shing Knowledge Institute, St. Michael’s Hospital, 30 Bond Street, Toronto, Ontario M5B 1W8 Canada; 8 University Health Network, Toronto General Hospital, 200 Elizabeth Street, Toronto, Ontario M5G 2C4 Canada; 9 Ottawa Hospital Research Institute, 725 Parkdale Avenue, Ottawa, Ontario K1Y 4E9 Canada; 10 School of Epidemiology, Public Health and Preventive Medicine, Faculty of Medicine, University of Ottawa, 451 Smyth Road, Roger-Guindon Building, Ottawa, Ontario K1H 8M5 Canada; 11 Department of Social Policy and Social Work, University of York, Heslington, York, YO10 5DD UK

**Keywords:** Scoping studies, Scoping reviews, Survey, Web-based, Research

## Abstract

**Background:**

Scoping studies (or reviews) are a method used to comprehensively map evidence across a range of study designs in an area, with the aim of informing future research practice, programs and policy. However, no universal agreement exists on terminology, definition or methodological steps. Our aim was to understand the experiences of, and considerations for conducting scoping studies from the perspective of academic and community partners. Primary objectives were to 1) describe experiences conducting scoping studies including strengths and challenges; and 2) describe perspectives on terminology, definition, and methodological steps.

**Methods:**

We conducted a cross-sectional web-based survey with clinicians, educators, researchers, knowledge users, representatives from community-based organizations, graduate students, and policy stakeholders with experience and/or interest in conducting scoping studies to gain an understanding of experiences and perspectives on the conduct and reporting of scoping studies. We administered an electronic self-reported questionnaire comprised of 22 items related to experiences with scoping studies, strengths and challenges, opinions on terminology, and methodological steps. We analyzed questionnaire data using descriptive statistics and content analytical techniques. Survey results were discussed during a multi-stakeholder consultation to identify key considerations in the conduct and reporting of scoping studies.

**Results:**

Of the 83 invitations, 54 individuals (65 %) completed the scoping questionnaire, and 48 (58 %) attended the scoping study meeting from Canada, the United Kingdom and United States. Many scoping study strengths were dually identified as challenges including breadth of scope, and iterative process. No consensus on terminology emerged, however key defining features that comprised a working definition of scoping studies included the exploratory mapping of literature in a field; iterative process, inclusion of grey literature; no quality assessment of included studies, and an optional consultation phase. We offer considerations for the conduct and reporting of scoping studies for researchers, clinicians and knowledge users engaging in this methodology.

**Conclusions:**

Lack of consensus on scoping terminology, definition and methodological steps persists. Reasons for this may be attributed to diversity of disciplines adopting this methodology for differing purposes. Further work is needed to establish guidelines on the reporting and methodological quality assessment of scoping studies.

**Electronic supplementary material:**

The online version of this article (doi:10.1186/s12913-016-1579-z) contains supplementary material, which is available to authorized users.

## Background

Scoping studies are a method to comprehensively synthesize evidence across a range of study designs. Scoping studies (or reviews) may be defined as “exploratory projects that systematically map the literature available on a topic, identifying key concepts, theories, sources of evidence and gaps in the research” [1]. Researchers may undertake a scoping study to examine the extent, range and nature of research activity, determine the value of undertaking a full systematic review, synthesize and disseminate findings, or identify gaps in existing literature [2].

Scoping studies have become increasingly popular in health research. The number of scoping studies has increased immensely in the past six years with over half of scoping studies published after 2012, demonstrating their growing potential to inform research agendas, and policy and practice recommendations [3]. Despite their increasing use and abundant promise for impact on practice, policy and research, no clear criteria exist to guide and evaluate scoping study rigor or reporting. While quality criteria and reporting guidelines exist for other methodological approaches, such as systematic reviews [4–6], and clinical practice guidelines [7], none have been established for scoping studies. Specific guidelines may enhance the reporting of scoping studies that address features related to breadth of purpose, evidence inclusion, and iterative nature of scoping studies that are unique from other syntheses.

In 2005, Arksey and O’Malley published a six-stage methodological framework for conducting scoping studies: identifying the research question, searching for relevant studies, selecting studies, charting the data, collating, summarizing and reporting the results, and consulting with stakeholders to inform or validate study findings [2]. In 2010, Levac et al. proposed recommendations, building on each stage of the scoping study framework, highlighting considerations for advancement, application and relevance of scoping studies in health research [8]. Additional recommendations for scoping study methodology have since been published, demonstrating the ongoing need and interest for advancement of this field [9–11]. Despite the progress to date, there remains no universal agreement on the definition or methodological steps for this approach [12].

We think it is important to address the methodological quality of this emerging method of evidence synthesis. Establishing a better understanding of current experiences including strengths and challenges of conducting scoping studies and exchanging knowledge and perspectives on terminology and methodological steps among stakeholders who share scoping study expertise is a first step to collaboratively advancing this methodology and providing a foundation for future development of methodological criteria in this field.

Our aim was to understand the experiences of, and considerations for conducting scoping studies from the perspective of clinicians, educators, researchers, graduate students and knowledge users with interest and experience in this methodology. Primary objectives were to 1) describe experiences conducting scoping studies including strengths and challenges, and 2) describe perspectives on terminology, definition and methodological steps. A secondary objective was to 3) discuss considerations in the conduct and reporting of scoping studies. Results will increase awareness of the methodological issues among researchers, clinicians and members of the knowledge user community to help inform future efforts on guidance of scoping study methodology.

## Methods

We conducted a web-based survey followed by a multi-stakeholder consultation meeting. For the purposes of this work, we used the term ‘scoping study’ throughout in accordance with the original Arksey and O’Malley Framework [2].

We conducted a cross-sectional web-based survey with a sample of researchers, health professionals, graduate students, policy makers and people living with chronic diseases in Canada, the United States and United Kingdom who possessed expertise and interest in scoping study and knowledge synthesis methodology. Individuals were invited to participate in the survey followed by a 2 day scoping study meeting to gain an understanding of the experiences, including strengths and challenges in conducting scoping studies, and obtain views on terminology, definition and methodological steps inherent to rigorous conduct of scoping studies.

We discussed the survey results in a multi-stakeholder scoping study consultation meeting with clinicians, academics, graduate students, representatives from community-based organizations, community members living with chronic illnesses, and policy and funding stakeholders. The goal of this meeting was to translate research evidence related to scoping study methodology and to establish priorities for the future development of criteria in the conduct and reporting of scoping studies [13].

We received ethics approval from the University of Toronto Health Sciences Research Ethics Board (Protocol Reference #: 31214).

### Participants

We administered a web-based self-administered questionnaire using a modified Dillman approach [14] and FluidSurveys [15] to all stakeholders who were invited to attend a 2 day scoping meeting in Toronto, Canada in June 2015.

We used a combination of snowball and purposive sampling to identify approximately 80 researchers, clinicians, graduate students, policy makers, and knowledge users in Canada, the United Kingdom (UK), and the United States. We asked researchers, knowledge users and collaborators for names and email addresses of individuals who they felt possessed an interest and expertise in scoping study methodology. Invited individuals also provided names and contact information of potential additional invitees. This process was critical in order to mobilize diverse participants who represent both developers and users of scoping studies in clinical practice, research and policy.

Given the consultative nature of this work, participants were likely persons whom members of the team previously worked with in an education, research or community-based capacity. In the case of the scoping study survey, completed questionnaires were considered implied consent. In the case of the meeting consultation, consent was based on the individual accepting the invitation and attending the meeting. All participants were invited to provide their name to be acknowledged in the manuscript (see Acknowledgements).

### Data collection

#### Scoping study questionnaire

We developed a scoping study questionnaire to describe the experiences, and views on scoping study terminology and methodological steps. The questionnaire was developed and pre-tested by four members of the scoping study team. The questionnaire took approximately 20 min to complete and included 22 items across six domains: 1) personal information; 2) experiences conducting scoping studies; 3) strengths and challenges of conducting scoping studies; 4) opinions on scoping study terminology, 5) key considerations for methodological steps and rigor, and 6) additional comments (see Additional file 1 – Scoping Study Questionnaire).

We sent an initial invitation email with an overview of the purpose of the scoping study survey and a link to the questionnaire. We sent three thank you / reminder emails at 1, 2 and 4 weeks after the initial email, thanking those who completed the questionnaire and asking those who had not responded to complete the questionnaire.

#### Scoping consultation meeting

The 2-day scoping study consultation meeting summarized the state of evidence in the field of scoping studies in the context of knowledge synthesis methodology. Sessions included a combination of structured presentations from speakers, a panel, and large and small group discussion segments to facilitate knowledge transfer and exchange of opinions for establishing a common definition and consensus surrounding methodological steps of scoping studies. Results from the web-based survey pertaining to terminology, definition and methodological steps were presented throughout the meeting followed by opportunities for discussion (see Additional file 2 – Scoping Study Meeting Agenda).

Perspectives on the scoping survey results were documented at the multi-stakeholder consultation through the following methods: 1) participants were asked to submit written considerations about the new and emerging issues in the field of scoping study methodology; 2) participants were encouraged to express their ideas as they pertained to the scoping study survey results related to terminology, definition, and methodological steps during the meeting discussion; and 3) six graduate student rapporteurs documented the discussion throughout the meeting. Responses on the questionnaire included information on the experiences, strengths and challenges of conducting scoping studies (objective #1). Collectively, data pertaining to perspectives and recommendations derived from the survey questionnaire and meeting sources provided the foundation for perspectives on terminology, definition and methodological steps (objective #2) and identifying key considerations for the conduct and reporting of scoping studies (objective #3).

### Analysis

*Scoping Study Questionnaire*: We calculated the view, participation and completion rates of the online questionnaire [16]. We analyzed questionnaire data using descriptive statistics using frequencies and percent (categorical items) and median and interquartile ranges (continuous items). We analyzed open ended response items using content analysis [17]. Three members of the core research team independently read the open-ended questions from the questionnaire to obtain an overall impression of the responses; and met to discuss overall impressions (KKO, HC, DL).

*Scoping Study Meeting Rapporteur Notes*: We analyzed the rapporteur data capturing the discussion at the scoping study meeting using content analysis [17]. Two members of the team independently read the rapporteur notes to obtain an overall impression of the reflections; and met to discuss overall impressions (KKO, LW). Rapporteur notes were independently coded by two members of the team (KKO, LW). Coding involved highlighting words or captions that captured key thoughts and concepts related to scoping study methodology. These codes were then clustered into broader categories that were collectively used to supplement the perspectives on terminology, definition and methodological steps and inform the key considerations for conduct and reporting of scoping studies.

The three reviewers for the questionnaire data (KKO, HC, DL) and two reviewers for the rapporteur summaries (KKO and LW) met twice to discuss the findings from the content analysis and six authors (KKO, LW, DL, HC, LB, AN) reviewed a preliminary version of the key considerations for refinement.

## Results and discussion

Of the 83 invitations, 63 (76 %; 63/83) viewed the questionnaire, and 54 (65 %; 54/83) completed the questionnaire [16]. The majority of respondents self-identified as researchers, coordinators, or managers (54 %), followed by graduate students (24 %), knowledge translation brokers (7 %), educators (4 %), community members (4 %), clinicians (2 %), policy makers (2 %), librarians (2 %), and consultants (2 %). Most respondents were from Canada (92 %; *n* = 50), with two participants from the UK (4 %) and two from the United States (4 %).

The scoping meeting was attended by 48 participants comprised of researchers (42 %; 20/48), graduate student trainees (29 %; 14/48), educators (13 %; 6/48), service providers (8 %; 4/48); community members (2 %; 1/48); and other professions (consultant, civil society representative and information specialist) (6 %; 3/48). The majority of meeting participants were affiliated with universities or academic institutions (58 %; 28/48), followed by community-based, not-for-profit, knowledge broker/translation, and government organizations (23 %; 11/48), research organizations (13 %; 6/48), and hospitals (6 %; 3/48). Most participants were from Canada (94 %; *n* = 45), with two from the United States (4 %), and one from the UK (2 %). Given our recruitment strategy was primarily through snowball sampling, participants were knowledgeable or had an interest in the field of scoping studies.

We report the findings as derived from the scoping study questionnaire related to experiences, strengths and challenges of conducting scoping studies (objective #1), followed by collective interpretations from the survey questionnaire and meeting consultation as they relate to terminology, definition and methodological steps (objective #2). We then reflect on key considerations that emerged from the scoping questionnaire and meeting consultation for moving forward (objective #3).

### Experiences, strengths and challenges of conducting scoping studies

#### Experience with scoping studies

The majority of questionnaire respondents (85 %) had experience engaging in a scoping study with most (87 %) having completed at least one scoping study and almost half having had a scoping study published or in press in a peer-reviewed journal (46 %; 25/54) (Table 1). Roles ranged from conceptualizing and refining the research question, oversight, conducting literature searches / reviews, scanning titles and abstracts to determine study inclusion, data extraction, synthesizing the evidence, writing-up, and disseminating findings. Most respondents engaged in scoping studies to examine the extent, range, nature of research activity (91 %), to map a body of evidence, and to identify gaps in the field; which was similarly documented as common purposes for conducting scoping studies [11] (Table 1).

**Table 1:** Experiences with Scoping Studies among Scoping Survey Respondents (*n* = 54 participants)

Scoping study experience	Number (%)
Ever involved in conducting a scoping study	46 (85 %)
Engaged in a stakeholder consultation as part of the scoping study process	19/46 (41 %)
Ever published a scoping study in a peer-reviewed journal	
Published	22 (41 %)
In Press	3 (6 %)
In Preparation	9 (17 %)
No	20 (37 %)
Number of Scoping Studies Completed	
None	7 (13 %)
1	14 (26 %)
2	8 (15 %)
3	9 (17 %)
4	6 (11 %)
5 or more	10 (18 %)
Purpose for Conducting a Scoping Study	
To determine the extent, range, nature of research activity	49 (91 %)
To identify research gaps in existing literature	46 (85 %)
To identify and summarize research evidence on a topic	45 (83 %)
To summarize and disseminate research findings	35 (65 %)
To determine value of undertaking a full systematic review	24 (44 %)
Other (e.g. gather ideas for educational strategies, develop evidence-based recommendations, to establish recommendations for future research, inform program development, academic requirement, inform policy makers, conduct review of policies, identify models of care)	11 (20 %)
Amount of Time Allocated to Conduct One Scoping Study	
0–3 months	7 (14 %)
6 months or less	9 (18 %)
6–12 months	28 (49 %)
> 1 year	6 (12 %)
Not applicable or ‘it depends’	4 (8 %)
Amount of Time it Actually Took to Conduct One Scoping Study	
0–3 months	6 (11 %)
6 months or less	6 (11 %)
6–12 months	21 (39 %)
> 1 year	11 (20 %)
Not applicable or ‘it depends’	10 (18 %)
Had Funding to Support the Conduct and Reporting of the Scoping Study	24 (44 %)
Scoping Study Framework	
Number of Respondents Used a Published Methodology	35 (65 %)
Type of Scoping Study Methodology Used	
Arksey and O’Malley (2005) [2]	31 (57 %)
Levac et al. (2010) [8]	21 (39 %)
Davis et al. (2009) [18]	5 (9 %)
Armstrong et al. (2011) [19]	3 (6 %)
Other (e.g. Daudt et al. (2013) [9], Anderson et al. (2008) [20], Wilson (2010) [21], Centre for Reviews and Dissemination (CRD) [22], Bragge (2011) [23])	5 (9 %)

Of the 46 respondents (85 %) who were involved in a scoping study, 19 (41 %) had engaged in a stakeholder consultation, 7 (37 %) of which went through Research Ethics Board (REB) review for the process. Reasons for pursuing REB review were because investigators intended to publish results and/or conducted interviews with ‘experts’. Respondents chose not go through REB review when the scoping study was conducted by a community organization, when there was no formal REB process available, when respondents did not plan to publish in an academic journal, when the scoping study was done to inform a systematic review, or the consultation involved stakeholders as part of a steering committee who provided feedback throughout the study. Finally, the majority of participants utilized a framework opposed to only half of investigators in a previous review suggesting there is increasing appetite for adopting a more rigorous approach to this methodology [11].

#### Strengths and challenges of scoping studies

**Fig. 1:**
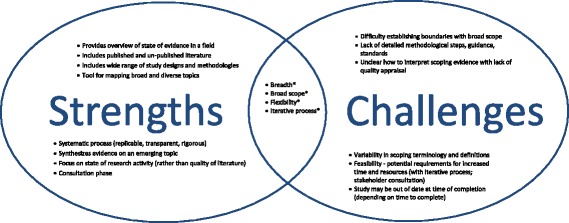
Strengths and Challenges of Scoping Studies. *characteristic that may be considered both a strength and challenge of scoping studies

Many scoping study strengths were dually identified as challenges (Fig. 1). Respondents described the strengths of scoping studies in relation to their ability to provide an ‘*overview of the state of evidence in a field’* their ‘*breadth’*, ‘*broad scope’* and ‘*tool for mapping broad and diverse topics’* to inform future work. Strengths associated with scoping study methodology included their flexibility*,* inclusion of published and unpublished (grey) literature, inclusion of literature with a wide range of study designs and methodologies, and the potential to combine qualitative and quantitative synthesis approaches. Respondents highlighted the systematic process (replicable, transparent, rigorous) that provides the ability to explore and synthesize evidence on a newly emerging topic (or topic with little published evidence), using an approach that is ‘*not as rigid as systematic reviews’*. Additional strengths included the ability for scoping studies to focus on the state of research activity rather than evaluate the quality of existing literature, the ability to assist policy makers to make evidence-informed decisions, and engagement of stakeholders with expertise in a given content area throughout all stages of the process.

Challenges dually identified as strengths of scoping studies included the flexibility, broad scope and inclusion of grey literature which posed challenges when trying to establish boundaries to the study scope (Fig. 1). One challenge included the variability in terminology and unclear definitions of concepts of interest, which made identifying the study scope difficult. The flexible and iterative process of defining (and redefining) the research question, search strategy and selection criteria required increased time and resources which was difficult to ascertain at the beginning of the study. The broad scope combined with lack of clarity around boundaries left some with an overwhelming amount of data, posing challenges for feasibility as scoping studies were often time-consuming and took longer than originally anticipated. This could place a scoping study at risk of quickly becoming out of date. Some respondents expressed challenges with the utility of a final product or outcome that was sometimes uncertain at the outset of the study: *“Sometimes it feels like a lot of work just to identify gaps or determine that more research is needed.”*

Respondents expressed challenges with the lack of detailed steps, guidance, standards, or methodology (particularly if and how to synthesize data from selected studies). They described the need for more clarification about the need and steps associated with the consultation phase. Some described difficulty finding unpublished (or grey) forms of evidence, synthesizing evidence from diverse sources with different types of data, diversity of reviewers or investigators involved, and lack of clarity of how to conduct the synthesis (or analysis) leaving some with an overwhelming amount of data if the scope was broad; and the lack of quality appraisal that resulted in the need for caution in interpretation. Some described challenges with the feasibility conducting the stakeholder consultation. Finally, respondents described challenges of reporting results succinctly, and the difficulty publishing scoping studies with limitations in journal word counts.

#### Scoping terminology

The majority of respondents preferred the term ‘scoping review’ (68 %) over ‘scoping study’ (11 %) with remaining respondents unsure or having no opinion suggesting either term could be appropriate, depending on the study (20 %) (Table 2).

**Table 2:** Scoping Study Terminology Preferences from Responses to the Scoping Study Questionnaire

Terminology preference	Example quotations
Scoping review	•“It is a review of the literature in this area similar to a systematic review. A study seems reserved from primary research with study participants.” • “Seems to me that the term review is more specific than study, and that’s what it seems to be: a review of the existing literature.” • “The name should reflect that standard review methods are used: search strategy, selection, data abstraction, and analysis (even if only descriptive). Reviews are still studies. Are some scoping studies not reviews?” • “The method is a form of a review of primary literature. Some scoping reviews gather primary data in the form of a stakeholder consultation but that is for the purpose of directing the synthesis and that alone does not provide a rationale for using the term ‘study’. We can limit terminology issues by choosing one term and of the two, study or review, review is a more accurate description of what it is.” • “The methodology of the scoping review is consistent with methods of evidence synthesis such as systematic reviews and meta-analysis methods, thus using the term ‘review’ defines the methodology more clearly than ‘study’.” • “The term “review” aligns with the purpose of the research (that at least I was aiming to use the methodology for). It was to review what was published in the literature, and to do so in a somewhat systematic way. Although it technically is a “study”, calling it a “review” seems to be more precise in my mind.”
Scoping study	• “Takes into account the analysis (thematic) component which differs from mere summary or synthesis in other types of reviews.” • “Use of the term scoping review positions this type of study in a hierarchical relationship with systematic review. Scoping studies have distinct goals and thus should be viewed as distinct entities and not as “less than” systematic reviews.” • “Additionally, the intent is not only to review the literature but to conceptualize it in a manner that speaks to the research question. I do not feel that the term ‘review’ covers the extent of analytic work of this type of study.” • “Clearer language. The use of the term “study” would also help to move away from scoping review, which seems to have become a catch-all term for everything that is not a systematic review.” • “Encompasses the entire methodology - the literature (or evidence) review phase as well as the consultation phase. It is also the original term coined by Arksey and O’Malley.”
Unsure; no opinion; depends on the study	• “I see advantages and disadvantages to both. I need better understanding of methodology to better answer the question.” • “It should be either, depending on whether or not something other than a review (e.g. a scoping survey) is carried out.”

Respondents who preferred the term ‘scoping review’ tended to focus on the literature review component of the methodology stating ‘review’ is “*a more accurate description*”, “*consistent with methods of evidence synthesis such as systematic reviews and meta-analysis methods”*. As one respondent stated:

*“the term “review” aligns with the purpose of the research….to review what was published in the literature, and to do so in a somewhat systematic way. Although it technically is a “study”, calling it a “review” seems to be more precise in my mind.”*

Respondents who preferred ‘scoping study’ did so because they “*take into account the analysis (thematic) component which differs from the mere summary or synthesis in other types of reviews”*. Another participant stated his/her preference in relation to the analytical difference between scoping studies and other types of knowledge syntheses:

*“Scoping studies have distinct goals and thus should be viewed as distinct entities and not as “less than” systematic reviews…. the intent is not only to review the literature but to conceptualize it in a manner that speaks to the research question. I do not feel that the term ‘review’ covers the extent of analytic work of this type of study.”*

Others felt that the term ‘scoping study’ provided clear language that helps distinguish them from ‘scoping reviews’ that have become a “*catch-all term for everything that is not a systematic review”*. Finally, scoping study terminology was preferred by those who felt the term ‘study’ encompassed the entire methodology, the literature (or evidence) review phase as well as the consultation phase. It was also acknowledged as the term coined by Arksey and O’Malley, the developers of the original Scoping Study Framework.

#### Defining features of scoping studies

Respondents described defining features of scoping studies, many of which were similar to strengths of the methodology (Table 1). Participants described the distinction of scoping studies from other forms of knowledge syntheses such as literature reviews, systematic or rapid reviews: “*[scoping studies] are not as rigorous as systematic reviews but more distinctive in methodology” and are “less likely to be strongly influenced by opinion than a traditional literature review”.*

Others struggled to distinguish scoping studies from other forms of syntheses as one participant stated:

*“This is challenging to distinguish. In many aspects scoping reviews are similar to systematic reviews in methodology with the capture of the broad research question, broad eligibility criteria, no quality assessment and consultation exercise.”*

Given the similarities in comprehensiveness and systematic nature, *“the difference in method is not as great as some might think”.* Similarly another participant did not perceive there to be distinctions between scoping studies and other syntheses:

*“I do not think that there is anything inherently distinct about scoping study methodology…I think that the ‘scoping study phenomenon’ is the product of a methodological title being applied widely and loosely for reasons that are completely logical given the current context of health sciences research.”*

Overall, participants described similarities between scoping and systematic reviews in relation to transparency, rigor, and systematic nature, whereas the distinction between scoping and literature reviews were easier to distinguish with scoping studies taking on a more systematic and rigorous nature of methodology.

#### Methodological steps

The majority of respondents to the scoping questionnaire (90 %) agreed that scoping studies should involve grey literature, and 31 % (16/54) felt that quality assessments of individual included studies should become a part of scoping methodology. Some respondents felt that quality assessment depends on the nature of the research question; for example if a question pertained to effectiveness of an intervention then quality assessment may be critical opposed to the exploration or description of an emerging topic where there may be less formulated evidence in a field.

### Perspectives on terminology, definition, and methodological steps

The following results include collective interpretations of the survey results from the meeting consultation as they relate to terminology, definition and methodological steps.

#### Terminology

Despite our intention, no consensus was achieved related to scoping terminology. Two opposing views appeared to emerge from the meeting: individuals who align scoping *reviews* (note the terminology) with other types of systematic reviews, and those who view scoping *studies* as distinct from other reviews. This may explain the lack of consensus surrounding terminology, methodological steps and positivist versus constructivist approaches (associated with reviews versus studies, respectively) to scoping the field of evidence. Others felt the distinction between ‘study’ over ‘review’ was attributed to the higher analytical level of synthesis of data from existing evidence (rather than reporting frequencies of literature characteristics) as well as the consultation phase that requires primary data collection and qualitative approaches to analysis, which results in a new outcome or product that is relevant and meaningful to the field; examples include a framework of existing evidence, or recommendations for future research priorities.

While the majority of questionnaire respondents (68 %) preferred the term scoping review (over study), the meeting provided insight to the historical development of the Scoping Study Framework from the perspective of Arksey and O’Malley. The preference for scoping review seemed to emerge from the methodological steps referencing review of the literature, and feeling that ‘scoping review’ provided more legitimacy and rigor if considered in the same classification of systematic reviews, which have clear established steps and reporting criteria [24]. However, the term ‘scoping study’ was intentionally chosen to distance itself from the ‘systematic review’ specifically aimed at determining clinical effectiveness of an intervention, to broaden awareness and inclusion of other types of evidence beyond the randomized controlled trial which may be traditionally favored in systematic reviews [13]. Scoping studies are intended to map a field and expose gaps in evidence. Developers borrowed elements from systematic reviews such as scoping studies as rigorous, systematic, replicable, and transparent while adding elements such as the iterative or flexible nature of the steps, lack of quality appraisal and addition of the consultation phase. Nevertheless, results suggest scoping studies may have evolved where researchers view them as a form of knowledge synthesis, hence adopting the term ‘review’. Overall, the way in which we distinguish scoping studies from other forms of reviews within the broader context of syntheses will be important for moving forward.

Challenges adopting universal terminology and a definition may be a reflection of the complex and diverse reasons in which someone may adopt scoping methodology, the diversity of research questions which this methodology may address (i.e. Arksey and O’Malley discuss four possible purposes alone), and the uptake across diverse disciplines using the methodology.

Another challenge distinguishing scoping studies from scoping reviews may be attributed to the systematic nature which both approaches adopt. Most survey respondents believed scoping studies (and reviews) are carried out *systematically* and it is the *systematic* characteristic that differentiates scoping studies (or reviews) from literature reviews. However, both terms (study and review) affiliate with similar characteristics with respect to their systematic, comprehensive and transparent nature, which may lead to difficulty in establishing how scoping studies are distinguished from other types of reviews. Peters et al. expressed the importance of distinguishing between scoping reviews and ‘comprehensive’ systematic reviews that also rely on synthesizing evidence from different study designs [25]: ‘While in a scoping review the goal is to determine what *kind* of evidence is available on the topic and to represent this evidence by mapping or charting the data, comprehensive systematic reviews are designed to answer a series of related but still very specific questions’ (Page 8) [25]. Scoping studies involve mapping the evidence describing the quantity, design and characteristics in a field opposed to systematic reviews which tend to address a specific narrowly defined research question [23]. This further highlights the importance of choosing the synthesis approach (and terminology) that best addresses the original research question. For instance, scoping studies may be better suited to exploratory questions in emerging fields that involve complex multi-factorial interventions with less high level randomized controlled trial evidence (e.g. rehabilitation), or areas with an abundance of grey but relevant literature that has not traditionally been taken up in a given field (e.g. policy). For example, scoping studies have been used to explore the emerging field of HIV and aging [26]. Authors characterized the literature describing the impact of aging on the health of older adults living with HIV (e.g. types of study design, participants, interventions (if applicable), health care domains addressed) and provided recommendations for future HIV and aging research [26].

#### Definition

Meeting participants generally agreed on a working definition of scoping studies as proposed by Colquhoun et al. [12] adapted to: ‘*a scoping study or scoping review is a form of knowledge synthesis that addresses an exploratory research question aimed at mapping key concepts, types of evidence, and gaps in research related to an emerging area or field by systematically and iteratively searching, selecting, summarizing and potentially synthesizing existing knowledge*.’

Debate emerged over the extent to which data are summarized or synthesized in scoping studies, whether synthesis is a defining characteristic of scoping studies and whether this makes it distinct from a literature review. For instance, the step of ‘collating the data’ could span the spectrum of pure counting of characteristics (descriptive) to a more analytical (thematic or content analysis) for variables of interest such as characteristics of interventions, outcomes, and authors conclusions. Debate also ensued over whether scoping studies should always yield recommendations for practice and policy, which require a higher level of synthesis and interpretation, and whether recommendations should only be developed after the formal consultation phase.

Overall, results from the survey and multi-stakeholder consultation provided rich insight into the views and perspectives of scoping terminology, definition and methodological steps providing further insight into why a lack of consensus may exist. The diversity of fields increasingly adopting this methodology may offer some explanation to the challenges seen to date in adopting a universal term and definition. Nevertheless, for this methodology to maintain the rigor which it aspires to achieve and sustain, it is critical we consider adopting clear and consistent language that may be transferrable across disciplines.

#### Methodological steps

Overall, meeting participants viewed scoping studies as systematic, rigorous and transparent, while allowing for flexibility of process. The following features emerged as distinct characteristics for each of the methodological steps: 1) *research question and scoping purpose* (exploratory, mapping, breadth); 2) *search strategy and study selection* (iterative, inclusive of grey literature and diverse study designs), 3) *charting collating and summarizing* (involves no quality assessment of included studies, may be iterative, synthesis could range from descriptive (counting characteristics of studies) to analytical (syntheses of qualitative and quantitative data involving thematic or content analysis) and 4) *consultation phase* conducted after the literature review (emerging from the field of qualitative research that involves consultation with knowledge users and community members to address the research question; an essential form of knowledge transfer and exchange).

While ongoing consultation may occur in other types of studies or reviews (providing expertise, context and interpretation), the consultation phase in scoping studies was viewed as a formalized and distinct phase involving key informants and/or stakeholders with expertise in the field of inquiry. The consultation phase in scoping studies, if undertaken, requires formal recruitment, data collection, qualitative approaches to analysis and interpretation, and ethics board review. Consultation goes beyond integrated or end-of-study knowledge translation and exchange and involves acquiring new information, enabling investigators to formally present findings from the literature synthesis, obtaining further experiential evidence and perspectives related to the scope of inquiry, and offering interpretations and considerations for the broader field. These characteristics provide a foundation from which to support our above proposed scoping study definition and will help to inform the development of future reporting guidelines.

### Key considerations in scoping study methodology

To our knowledge this is the first study to explore experiences and opinions on scoping study methodology from the perspectives of researchers, clinicians, community members, representatives from community-based organizations, and policy stakeholders with expertise and/or interest in this methodology. This work directly builds on earlier work in an attempt to offer considerations related to terminology, definition and guidance on the methodological steps and rigor for the conduct and reporting of scoping studies [8, 9, 11, 12].

We will now discuss key considerations for engaging in scoping study methodology that emerged from the survey and multi-stakeholder consultation with researchers, clinicians, knowledge users and policy stakeholders.

*Ongoing debate of scoping terminology, definition, and purpose:* Respondents expressed the need to develop clarity and consensus on terminology, definition, and purpose of conducting a scoping study. This will help to indicate when a scoping study is appropriate (and when it is not), and provide a foundation for developing detailed descriptions of each methodological step in conducting and reporting guidance for scoping studies. In the future we might consider whether clarity around the conceptual meaning of the terms ‘scoping study’ versus ‘scoping review’ may help to distinguish where this methodology lies on the spectrum of knowledge syntheses. Scoping studies also may be referred to either a map or syntheses, depending on whether they *describe* the characteristics versus *synthesize* the literature [27]. We did not specifically aim to determine how scoping study definitions and methodologies differ or align with other types of syntheses. This is an important focus of future work. Distinguishing ways in which scoping studies (or reviews) differ from other review methodologies for key dimensions including aims and approaches, structure and components; and breadth and depth, provides a strategy for clarifying the definition and purpose of this methodology [28].

*Clarity of concept of interest and methodological steps*: Clearly defining the concept of interest can help to establish boundaries for the scope of inquiry, search strategy and inform the study selection process. Specific steps respondents felt required more clarity and instruction included i) how to engage stakeholders throughout the process, ii) clarity around the consultation phase, iii) more detail on stage five of collating, summarizing and reporting the results, specifically how to go about the synthesis and reporting of grey literature, and how to synthesize, and report a combination of quantitative and qualitative synthesis techniques, and iv) adding a methodological step specific to knowledge translation of scoping study results. Piloting the process for study selection and data extraction can help to ensure universal understanding of the construct of interest, study selection criteria, and characteristics of interest for extraction among members of the team [8]. Recently published guidelines provide detailed steps on how to define these concepts in a study and how they can help to inform the selection criteria and search strategy [25, 29]. Further insight is needed for ways to optimally present study findings to best represent the ‘mapping of evidence’ (e.g. framework, tables, and figures) within the context of journal requirements for publication.

*Consultation phase:* Respondents articulated the need to consider the consultation phase as distinct but complementary to ongoing consultation throughout the scoping process. The integrated consultative approach often is informal and does not require formal forms of data collection or analysis; the stakeholder is a member of the scoping team. Alternatively, consultation is a formal stage of the Arksey and O’Malley Framework whereby experts and key informants are formally consulted on the results of the literature review and provide further insight into experiential evidence, interpret what is found in the literature (strengths and gaps), and provide recommendations for moving forward [2]. The consultation phase involves sharing findings from the literature review and gaining additional expertise or perspectives on the construct of interest. In most cases the consultation phase will require a formal phase of recruitment and data collection requiring REB review. While increasing time and complexity, the consultation phase has the potential to enhance the quality and relevance of findings to the field [8].

*Methodological quality assessment.* Absence of methodological quality assessment was considered a distinct feature of scoping studies as the aim of this approach is to map the evidence produced in a given field rather than seeking out the *best available evidence* to answer a question related to policy and practice [25]. However, some recommend quality assessment to determine study inclusion [9]. If the lack of quality assessment is a defining feature of scoping studies, does introducing this component by nature no longer define it as a scoping study? Participants in this study felt that absence of methodological quality assessment as a defining feature of scoping studies requires further discussion. Methodological quality may be of value in certain circumstances depending on the research question, specifically if it relates to determining the impact or effect of an intervention or program. In these instances, a systematic review may be more appropriate. Overall, the increasing number of types of knowledge syntheses can result in a lack of clarity when choosing (or not choosing) to undertake a scoping study. The research question, the context of the field, and the type and level of evidence available are important considerations for choosing a scoping study over an alternate form of knowledge synthesis.

These considerations pose further inquiry and examination in order to move the field forward with clarity and consensus surrounding terminology, definition and methodological steps. Strategies to enhance methodological rigour that emerged from this work similarly reflect those documented in the literature such as clearly justifying the use of scoping study methodology over other forms of knowledge syntheses, and articulating a clear and focused scoping purpose which directly links to the research question and study objectives [8, 25, 29]. In the absence of clear consensus, we recommend continuing to follow the steps as outlined in the Scoping Study Framework by Arksey and O’Malley [2] and supplemented by Levac et al. [8] and Daudt and colleagues [9], while considering newly published recommendations on the methodological protocol of conducting and reporting scoping studies [25, 29].

### Considerations for interpretation

This consultation was built on the foundational work by Arksey and O’Malley who established the original Scoping Study Framework [2]. Using a combination of survey methods followed by a multi-stakeholder consultation enabled developers and users of scoping studies to reflect and consider views of scoping study terminology, definition and steps after participants at a 2-day meeting which included a historical background and overview from Lisa O’Malley (LOM). Four authors are rehabilitation professionals who work in areas of complex chronic disease, hence people living with chronic disease were key knowledge users involved throughout this work. These authors developed an interest in scoping study methodology after each completed a scoping study in separate areas of rehabilitation and experienced similar challenges implementing the methodology. Scoping studies offered a method to comprehensively synthesize evidence across a range of study designs where a paucity of randomized controlled trials can exists in the field of rehabilitation and chronic illness.

Our approach was not without limitations. First, this is a small sample primarily from Ontario, Canada with a diversity of expertise in scoping studies; hence the perspectives are not fully representative of experts and people conducting scoping reviews nationally or internationally. Nevertheless, participants represented a range of perspectives from those involved in developing the original Framework, other reporting guidelines, and participating in studies offering diverse perspectives and experiences form which to draw these considerations. Future work may consider expanding representation from other countries where the role for scoping studies is emerging. Lastly, the paper provides broad considerations for engaging in scoping study methodology. Next steps will be for researchers, clinicians and knowledge users to conduct a broader consultation to further advance the terminology and definition, develop a methods manual, reporting guidelines and methodological quality criteria of this budding field.

## Conclusions

Overall we provide an overview of experiences, views on terminology, definition, methodological steps, and key considerations in the conduct and reporting of scoping studies. This work provides a foundation for researchers, clinicians and knowledge users to consider when thinking about using scoping study methodology. Results reflect the current state of the scoping study field as it evolves and is increasingly adopted by different disciplines. While a challenging time, it also reflects an exciting time and opportunities in which this methodology is being adopted by various disciplines. Further work is needed to clarify terminology and definitions in order to formulate methodological quality criteria for conducting and reporting the emerging field of scoping studies.

## Abbreviations

HIV, Human Immunodeficiency Virus; REB, Research Ethics Board.

## Additional files

Additional file 1: Scoping Study Questionnaire. (PDF 159 kb) Additional file 2: Scoping Study Consultation Meeting Agenda. (PDF 142 kb)
