# Supplementary material for: Advancing scoping study methodology: a web-based survey and consultation of perceptions on terminology, definition and methodological steps
Source: BMC Health Serv Res. 2016 Jul 26;16:305. doi: 10.1186/s12913-016-1579-z (PMC4962390; doi:10.1186/s12913-016-1579-z)
Supplement: Additional file 1: — Scoping Study Questionnaire. (PDF 159 kb) [file 12913_2016_1579_MOESM1_ESM.pdf]

Additional File 1: Scoping Study Questionnaire

Introduction

Scoping studies are an ideal method to more comprehensively synthesize evidence across a range of study designs from both published and grey literature in rehabilitation and chronic illness, with the aim of informing practice, programs and policy and providing direction to future research priorities. CIHR defines scoping studies (or reviews) as '*exploratory studies that systematically map the literature available on a topic, identifying the key concepts, theories, sources of evidence and gaps in the research*.' Researchers and clinicians may undertake a scoping study to examine the extent, range and nature of research activity, determine the value of undertaking a full systematic review, synthesize and disseminate findings, or identify gaps in existing literature.

Recommendations for scoping study methodology have since been published, however there remains no universal agreement on the definition or methodological steps for this approach. Forming partnerships and exchanging knowledge among researchers who share scoping study expertise is essential to collaboratively advance this methodology and address future research priorities in the field.

Establishing methodological criteria for conducting and reporting scoping studies will help researchers and clinicians strengthen the rigor in which scoping studies are undertaken, enhance the validity of evidence, and promote advances in best practices in health and rehabilitation for older adults with complex chronic disease. Establishing consensus on the methodological steps to scoping studies is a first step to informing the development of the future criteria.

What is the goal of this environmental scan?

To obtain an understanding of experiences and perspectives on the conduct and reporting of scoping studies. Specifically we are interested in learning about your experience (including strengths and challenges) conducting scoping studies, your views on scoping study terminology, and perspectives on methodological steps in conducting scoping studies. By summarizing this information and relaying it to team members at the meeting in June 2015, we will be able to collaboratively define "scoping study" and establish a consensus on the methodological steps for conducting scoping studies.

Who can participate?

As an invitee to the upcoming Scoping Study Meeting on June 8-9th, 2015, you have been identified as someone who has experience with scoping studies or has the potential to utilize scoping study evidence in your work. Regardless of your level of experience with scoping reviews, we would like to hear from you. We are interested in your views even if you cannot attend the meeting.

How will the information from this environmental scan be used?

Findings from this environmental scan will be used to inform the agenda for the upcoming Scoping Study Meeting on June 8-9th, 2015. Results from this environmental scan will be shared at the meeting and with everyone who participated in this environmental scan.

Additional File 1: Scoping Study Questionnaire

What is involved?

You will be asked to complete an online web-based questionnaire. The questionnaire includes items about your experience conducting scoping studies, thoughts on scoping study terminology and methodological steps. As an invitee to the Scoping Study meeting, you will be asked to provide contact information so that we can follow up with any further clarification as we finalize the meeting agenda. Results from the scan will be presented in aggregate form and individual respondents will not be identified in the presentation of the results.

How long will it take?

The questionnaire takes approximately 20 minutes to complete.

If I start the questionnaire, can I stop?

This questionnaire is voluntary. You can stop at any time. You do not have to answer all the questions. If you decide you do not want to complete the questionnaire, you can still attend the Scoping Study Meeting.

Why should I take part?

Your views will help us gain an overall understanding of experiences in conducting scoping reviews as well as strengths and challenges to conducting scoping reviews. Findings from the scan will directly inform the Scoping Study Meeting in Toronto in June 2015.

Who is conducting this environmental scan?

This scan is being done by a team of researchers, rehabilitation professionals and community members from the University of Toronto and St. Michael's Hospital. The Canadian Working Group on HIV and Rehabilitation (CWGHR) (www.hivandrehab.ca) is a collaborator in this work.

Who is funding this Scan?

This work is funded by the Canadian Institutes of Health Research (CIHR).

How can I get a summary of the study results?

We will present the results at the Scoping Study Meeting in June 2015. We will summarize the results in a report from the Scoping Study meeting of which the scan results will be included. Results from the environmental scan will be written up in the form of a manuscript for publication.

Who can I contact if I have questions?

If you have any questions please contact Ayesha Nayar, Research Coordinator at the University of Toronto, at ayesha.nayar@alum.utoronto.ca, or by phone 416-946-3935 or toll free 1-855-589-0249.

Advancing Scoping Study Methodology: A web-based survey and consultation of perceptions on terminology, definition and methodological steps

Additional File 1: Scoping Study Questionnaire

If you have any questions regarding your rights as a participant in this survey, you can also contact the Office of the Research Ethics of the University of Toronto at 416-946-3608.

To start the Scoping Study Environmental Scan, click on the Next>> button below to continue.

Instruction Page

The Environmental Scan Questionnaire has 6 sections with a total of 22 questions. .

To go through the questionnaire, you must use the "<<Previous" and "Next>>" buttons at the bottom of the screen. Please do not use Back or Forward arrows on your browser. Thank you for participating in this environmental scan. Click 'NEXT'

Section 1: Personal Information

Scoping studies (or reviews) are defined as 'exploratory studies that systematically map the literature available on a topic, identifying the key concepts, theories, sources of evidence and gaps in the research' (CIHR, 2010). Scoping studies can help to examine the extent, range and nature of research activity, determine the value of undertaking a full systematic review, synthesize and disseminate findings, or identify gaps in existing literature. For the purpose of this scan, we use the term scoping study throughout.
This section asks some background questions about you.

1) Please provide your contact information. (This information will help to inform planning for the meeting (e.g. establishing working groups with diversity based on position and place of work). Personal information will not be linked to the responses in the environmental scan and in the reporting of results.)
   a) Name:
   b) Email:
   c) Title / Position:
   d) Organization:
   e) Address:

2) What best describes your current position? (Please select one)
   - ☐ Researcher
   - ☐ Clinician
   - ☐ Educator
   - ☐ Service Provider
   - ☐ Student
   - ☐ Community Member
   - ☐ Policy maker
   - ☐ Volunteer
   - ☐ Funder
   - ☐ Other (Please specify)

Additional File 1: Scoping Study Questionnaire

3) Which of these <u>best</u> describes your place of work? (Please select one)
   - ☐ University (or other Academic Institution)
   - ☐ Hospital
   - ☐ Primary Care/General Practice
   - ☐ Service Provider Organization (community)
   - ☐ Research/Knowledge Production Organization
   - ☐ Other (Please specify)

<u>Section 2: Experience with Scoping Studies</u>

This section asks some questions about your experience with scoping studies.

4) Have you ever been involved in conducting a scoping study?
   - ☐ Yes
   - ☐ No

4A) You indicated in Question #4 that you have been involved in conducting a scoping study. Please describe your role. ____

4B) You indicated in Question #4 that you have been involved in conducting a scoping study. Did you engage in stakeholder consultation as part of the scoping study process?

- ☐ Yes
- ☐ No

4C) You indicated in Question #4B that you engaged in stakeholder consultation as part of the scoping study process. Did you go through Research Ethics Board (REB) review to complete the stakeholder consultation?

- ☐ Yes
- ☐ No

4D) Please explain why you did, or did not, go through REB review to complete the stakeholder consultation for the scoping study process. ___________________________

5) Have you been involved in reporting (publishing) a scoping study in a peer-reviewed journal?
   - ☐ Yes
   - ☐ No
   - ☐ In press
   - ☐ In preparation

6) How many scoping studies have you completed? (include those currently underway)

   ____

Additional File 1: Scoping Study Questionnaire

7) Overall, what was your purpose(s) or objective(s) for conducting a scoping study? Check 'Yes' or 'No' for each of the following:

	Yes	No	Unsure
a) To examine the extent, range and nature of research activity.	☐	☐	☐
b) To determine the value of undertaking a full systematic review.	☐	☐	☐
c) To summarize and disseminate research findings.	☐	☐	☐
d) To identify research gaps in the existing literature.	☐	☐	☐
e) To identify and summarize research evidence on a topic.	☐	☐	☐
f) Other (Please specify).	☐	☐	☐

8) Did you use a published methodology to complete the scoping study?

- ☐ No
- ☐ Yes
- ☐ Unsure

8A) You indicated in Question #8 that you used a published methodology to complete the scoping study. Which one did you use? (You may select more than one option).

- ☐ Arksey and O'Malley (2005)
- ☐ Levac et al (2010)
- ☐ Davis et al (2009)
- ☐ Armstrong et al (2011)
- ☐ Other-please specify

9) Approximately, how much time (in months) do (did) you allocate to conduct one scoping study?

- ☐ 0-3 months
- ☐ 6 months or less
- ☐ 6 months – 12 months (1 year)
- ☐ Over 1 year
- ☐ Other (Please explain)
- ☐ None – I have not conducted a scoping study

Additional File 1: Scoping Study Questionnaire

9A) How much time did it actually take (or is it taking) you to conduct one scoping study?

- ☐ 0-3 months
- ☐ 6 months or less
- ☐ 6 months – 12 months (1 year)
- ☐ Over 1 year
- ☐ Other (Please explain)
- ☐ None – I have not conducted a scoping study

10) Did (or do) you have funding to support the conduct of the scoping study?

- ☐ Yes
- ☐ No
- ☐ Not applicable – I have not conducted a scoping study

10A) You indicated in Question #10 that you did (or do) have funding to support the conduct of the scoping study. What was the name of the funding organization?

____

Section 3: Strengths and Challenges of Conducting Scoping Studies

This section asks your opinions on the strengths and challenges of conducting scoping studies.

11) What would you identify as **strengths** of scoping study methodology? Please list below.

-
-
-

12) What would you identify as the **challenges or limitations** of conducting a scoping study? Please list below.

-
-
-

13) What **strategies** could be used to address these challenges or limitations?

-
-
-

Additional File 1: Scoping Study Questionnaire

Section 4: Thoughts on Terminology- Scoping Studies vs. Scoping Review

This section asks about your views on scoping study terminology.

14) In your opinion, what should be the standardized term for this method of research?

- ☐ Scoping Study
- ☐ Scoping Review
- ☐ Other. Please specify. ____

14A) Please Explain: ____

____

Section 5: Considerations for Methodological Steps and Rigor

This section asks your opinion on the defining features of scoping studies (or reviews) and considerations for enhancing rigor.

15) In your opinion, what are the key defining features of a scoping study (or review)?

16) How are scoping studies distinct from other reviews (e.g. systematic reviews, literature reviews, bibliographic reviews, rapid reviews)?

17) Currently quality assessment of individual included studies is not a part of the formal scoping study methodology. Do you think that conducting quality assessments of individual included studies should become part of scoping study methodology?

- ☐ Yes
- ☐ No

17A) Please explain your reasoning for your answer in Question #17:
____

18) Should scoping studies involve grey literature?

- ☐ Yes
- ☐ No

Additional File 1: Scoping Study Questionnaire

19) What recommendations do you have for advancing the methodology of the scoping study approach; particularly in the health and rehabilitation research field?

-
-
-

<u>Section 6: Additional Comments</u>

This section asks about any final comments or recommendations for the upcoming Scoping Study meeting in June 2015.

20) What are the primary issues related to scoping study methodology that the Scoping Study Meeting should address?

-
-
-

21) Do you have any other comments, recommendations or issues about scoping studies (or reviews)?

22A) Would you like to be acknowledged in the final report and manuscript of scoping study meeting?

☐ Yes
☐ No

22B) If Yes, please indicate your name and affiliation of preference for the acknowledgement:

Name: ______________________________________ Affiliation: ______________________________________

Thank you for completing this questionnaire. We look forward to seeing those of you who will be attending the Scoping Study Methodology Meeting on June 8-9th, 2015 in Toronto, Ontario.

If you have any questions please contact Ayesha Nayar, Research Coordinator at the University of Toronto, at ayesha.nayar@alum.utoronto.ca, or by phone 416-946-3935 or toll free 1-855-589-0249.

Acknowledgements

The Meeting to Advance Scoping Studies Methodology is funded by a Planning Grant from the Canadian Institutes of Health Research (CIHR).

CIHR IRSC
Canadian Institutes of Health Research | Instituts de recherche en santé du Canada
