# Supplementary material for: Advancing scoping study methodology: a web-based survey and consultation of perceptions on terminology, definition and methodological steps
Source: BMC Health Serv Res. 2016 Jul 26;16:305. doi: 10.1186/s12913-016-1579-z (PMC4962390; doi:10.1186/s12913-016-1579-z)
Supplement: Additional file 2: — Scoping Study Consultation Meeting Agenda. (PDF 142 kb) [file 12913_2016_1579_MOESM2_ESM.pdf]

Additional File 2: Scoping Study Consultation Meeting Agenda

**Day 1 - The Scoping Experience, Terminology and Definition**		
Objectives:<br>1) To facilitate **knowledge transfer and exchange (KTE)** among researchers, clinicians and knowledge users about scoping study methodology.<br>2) Develop a **common definition of a 'scoping study'.**		
**Time**	**Topic**	**Speaker**
900-920	Welcome and Introductions<br>Why Methodological Quality Criteria for Scoping Studies?<br>Overview of Agenda and Objectives of the Meeting	Kelly O'Brien,<br>Heather Colquhoun,<br>Danielle Levac
920-1000	**Keynote Speaker Session #1 - Overview of Scoping Study Methodology –** Development of the Arksey and O'Malley Framework	*Keynote Speaker*<br>Lisa O'Malley (UK)
1000-1020	**Enhancing Scoping Study Methodology**<br>**Building on the Arksey and O'Malley Framework**	Danielle Levac
1020-1040	**Scoping review of Scoping Reviews**	Mai Pham
1040-1100	**Break**	
1100-1230	**Panel Session - Scoping Studies – Experiences, Strengths, Benefits and Challenges and Strategies for Moving Forward**<br><br>**Questions and Facilitated Large Group Discussion**	Session Introduction:<br>Kelly O'Brien<br><br>Panelists and Moderator
1230-130	**Lunch**	
130-150	**Scoping Reviews: Where Do They Sit on the Spectrum of Knowledge Syntheses?**	Andrea Tricco
150-210	**Introduction to Establishing a Common Definition and Terminology of Scoping Studies / Reviews**	Danielle Levac,<br>Kelly O'Brien
210-315	**Small Group Breakout Session –Strengths and Challenges of Scoping Studies & Developing a Definition and Terminology**	Small Groups
315-330	**Break**	
330-430	**Reporting Back to the Large Group – Large Group Discussion**	Kelly O'Brien,<br>Danielle Levac
430-500	**Summary and Recommendations for Next Steps**<br>**Wrap Up and Evaluation of Day 1**	Lisa O'Malley

Additional File 2: Scoping Study Consultation Meeting Agenda

Day 2 – Methodological Steps to Scoping Studies

Objectives:
1) To establish consensus on the **methodological steps** for conducting scoping studies.
2) To **determine the process** for the development, pilot testing and validation of the quality criteria;
3) To develop an **international collaborative on scoping study methodology.**

Time	Topic	Speaker
900-910	**Welcome and Review of Day 1**<br>**Overview of Day 2 Agenda & Review of the Common Characteristics of a Definition based on the Day 1 Discussion**	Heather Colquhoun
910-950	**<u>Keynote Speaker Session #2:</u> Results from a Knowledge Synthesis on Scoping Studies and Methodology Quality**	*Keynote Speakers*<br>Wasifa Zarin &<br>Erin Lillie
950-1030	**<u>Keynote Speaker Session #3</u> –What is EQUATOR and What Are the Considerations for Developing Health Research Reporting Guidelines**	*Keynote Speaker*<br>David Moher
1030-1045	**Break**	
1045-1230	**Introduction: Establishing Methodological Steps for Conducting Scoping Studies (Modified Delphi and Large Group Discussion)**	Heather Colquhoun,<br>Andrea Tricco
1230-130	**Lunch**	
130-300	**Establishing Consensus on the Methodological Steps for Conducting Scoping Studies – Large Group Discussion Continued - Delphi**	Heather Colquhoun,<br>Andrea Tricco
300-315	**Break**	
315-345	**Bringing it all Together – Next Steps - Developing the Plan for the Development of the Methodological Criteria, Future Pilot Testing and Validation**	Andrea Tricco
345-415	**Knowledge Translation and Exchange Strategy – Translating the Methodological Steps of Scoping Studies &**<br>**Sustainability of a Network of Interest in Scoping Studies**	Heather Colquhoun<br>Kelly O'Brien,<br>Danielle Levac
415-430	**Wrap-Up, Acknowledgements and Evaluation**	Heather Colquhoun,<br>Danielle Levac,<br>Kelly O'Brien

Acknowledgements

The Meeting to Advance Scoping Studies Methodology was funded by a Planning Grant from the Canadian Institutes of Health Research (CIHR).

CIHR IRSC
Canadian Institutes of Health Research
Instituts de recherche en santé du Canada
